# Trichodermin (12,13-ep­oxy­trichethec-9-en-4β-yl 4-fluoro­benzoate)

**DOI:** 10.1107/S1600536810028461

**Published:** 2010-07-24

**Authors:** Xu-hui Xu, Zong-cheng Wang, Jing-li Cheng, Yong Zhou, Jin-hao Zhao

**Affiliations:** a Institute of Pesticide and Environmental Toxicology, Zhejiang University, Hangzhou 310029, People’s Republic of China; b College of Pharmaceutical Sciences, Zhejiang University of Technology, Hangzhou 310032, People’s Republic of China

## Abstract

In the title trichodermin compound (systematic name: 12,13-ep­oxy­trichothec-9-en-4β-yl 4-fluoro­benzoate), C_22_H_25_FO_4_, the five-membered ring displays an envelope conformation, whereas the two six-membered rings show the different conformations, *viz*. chair and half-chair. As for the seven-membered ring, the dihedral angle between the mean planes formed by the four C atoms of the envelope unit and the three C and one O atoms of the six-membered chair is 68.67 (2)°; these two mean planes are nearly perpendicular to the ep­oxy ring with angles of 87.97 (2)and 88.14 (2)°, respectively.

## Related literature

The endophytic fungi Trichoderma taxi *sp*. nov. can produce a compound with fungicidal activity, Trichodermin (Zhang *et al.*, 2007 ▶), which is a member of the 4β-acet­oxy-12,13-ep­oxy­trichothecene family (Nielsen *et al.*, 2005 ▶). For a related Trichodermin structure, see: Chen *et al.* (2008 ▶). For the structures of Trichodermin derivatives, see: Cheng *et al.* (2009 ▶); Zhao *et al.* (2010 ▶). For the extinction correction, see: Larson (1970 ▶).
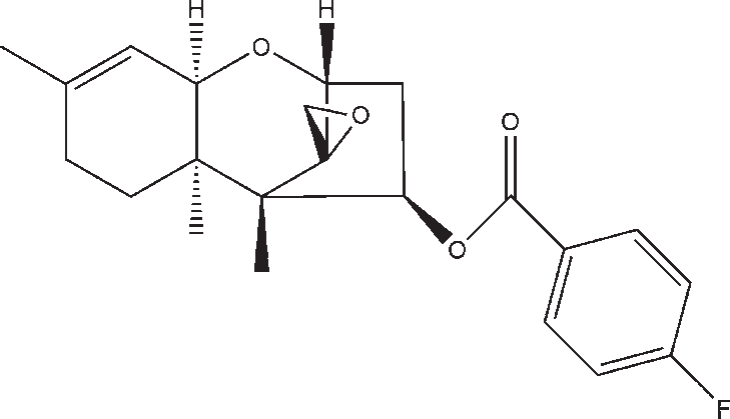


## Experimental

### 

#### Crystal data

C_22_H_25_FO_4_
*M* *_r_* = 372.42
Orthorhombic, 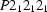

*a* = 8.1643 (4) Å
*b* = 9.9979 (4) Å
*c* = 23.6503 (9) Å
*V* = 1930.48 (14) Å^3^
*Z* = 4
Mo *K*α radiation
μ = 0.09 mm^−1^
*T* = 296 K
0.49 × 0.38 × 0.28 mm

#### Data collection

Rigaku R-AXIS RAPID/ZJUG diffractometer
Absorption correction: multi-scan (*ABSCOR*; Higashi, 1995 ▶) *T*_min_ = 0.956, *T*_max_ = 0.974
16756 measured reflections
2183 independent reflections
1760 reflections with *I* > 2σ(*I*)
*R*_int_ = 0.030

#### Refinement

*R*[*F* ^2^ > 2σ(*F* ^2^)] = 0.034
*wR*(*F* ^2^) = 0.086
*S* = 1.00
2183 reflections
248 parameters
H-atom parameters constrained
Δρ_max_ = 0.16 e Å^−3^
Δρ_min_ = −0.16 e Å^−3^

### 

Data collection: *PROCESS-AUTO* (Rigaku, 2006 ▶); cell refinement: *PROCESS-AUTO*; data reduction: *CrystalStructure* (Rigaku, 2007 ▶); program(s) used to solve structure: *SHELXS97* (Sheldrick, 2008 ▶); program(s) used to refine structure: *SHELXL97* (Sheldrick, 2008 ▶); molecular graphics: *ORTEP-3 for Windows* (Farrugia, 1997 ▶); software used to prepare material for publication: *WinGX* (Farrugia, 1999 ▶) and *PLATON* (Spek, 2009 ▶).

## Supplementary Material

Crystal structure: contains datablocks global, I. DOI: 10.1107/S1600536810028461/si2275sup1.cif

Structure factors: contains datablocks I. DOI: 10.1107/S1600536810028461/si2275Isup2.hkl

Additional supplementary materials:  crystallographic information; 3D view; checkCIF report

## Figures and Tables

**Fig. 1.**
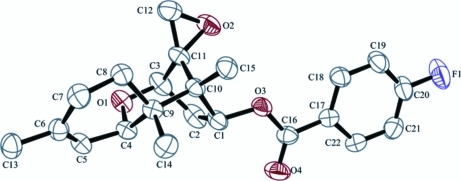
The molecular structure of (I), showing the atom-labelling scheme. Displacement ellipsoids are drawn at the 40% probability level.

## supplementary crystallographic information

### Comment

The endophytic fungi Trichoderma taxi *sp*. nov. from Taxus mairei S.Y.Hu can produce a compound with fungicidal activity-Trichodermin (Zhang *et al.*, 2007), which is a member of the 4β-acetoxy-12,13-epoxytrichothecene family (Nielsen *et al.*, 2005). Bioassays showed Trichodermin strongly inhibited Rhizoctonia solani and Botrytis cinere. Furthermore, addition of bromine at the C9=C10 double bond led to the absolutely loss in antifungal activity (Zhao *et al.*, 2010). 4β Conformation and its acetate are key moieties for its bioactivities (Zhang *et al.*, 2007; Cheng *et al.*, 2009). Therefore, in order to develop novel, safe and potential microbial pesticides, the 4β substituted phenyl esters such as the title compound were designed and synthesized. Its molecular structure is shown in Fig. 1. In the molecule, the five membered ring displays an envelope conformation with C11 atom at the flap position 0.695 (3) Å out of the mean plane formed by the other four atoms. The two six-membered rings display different conformations. The O1-containing ring displays a chair conformation. Whereas the C6-containing six-membered ring displays a half-chair conformation. As for the seven-membered ring, the dihedral angle between the mean planes formed by C1,C2,C3,C10 and C3,O1,C9,C10 is 68.67 (2) °, which are nearly perpendicular to the epoxy ring with angles of 87.97 (2) and 88.14 (2) °, respectively.

### Experimental

In a flask, 4β-hydroxy-12,13-epoxytrichethec-9-ene 2.50 g (10 mmol) were introduced with 40 ml of absolute methylene chloride, and then 1.34 g (11 mmol) of triethylamine was added. After the mixture was dissolved, a solution of 1.73 g (11 mmol) of 4-fluorobenzoyl chloride in 10 ml of absolute methylene chloride was added dropwise in 20 min at 273–278 K. After stirring for 2 h, the reaction solution was washed with 1% HCl solution, sat. NaHCO_3_, and sat. NaCl solution respectively. After dried with sodium sulfate, the solution was concentrated. The residue was crystallized and purified with 95% ethanol to colourless blocks. The 1H NMR,ESI-MS data testified the title compound's structure. 1*H*-NMR (500 MHz, CDCl_3_, ppm): 8.09–7.09 (4*H*, m, H-4), 5.80–5.77 (1*H*, m, H-4), 5.44–5.43 (1*H*, m, H-10), 3.89 (1*H*, d, J=5.5 Hz, H-2), 3.68 (1*H*, d, J=5.5 Hz, H-11), 3.17 (1*H*, d, J=4.0 Hz, H-13), 2.87 (1*H*, d, J=4.0 Hz, H-13), 2.67–2.63 (1*H*, m, H-3), 2.15–2.11 (1*H*, m, H-3), 2.03 (2*H*, s, H-8), 2.01–1.95 (1*H*, m, H-7), 1.73 (3*H*, s, H-16), 1.46–1.43 (1*H*, m, H-7), 0.98 (3*H*, s, H-14), 0.792 (3*H*, s, H-15); ESI-MS: 372 (*M*+H)+, (100%).

### Refinement

In the absence of significant anomalous scattering effects, Friedel pairs were merged; the absolute configuration was not determined. The H atoms were geometrically placed (C–H = 0.93–0.98 Å) and refined as riding with *U*_iso_(H) = 1.2*U*_eq_(C).

### Crystal data

**Table:**

C_22_H_25_FO_4_	*F*(000) = 792
*M**_r_* = 372.42	*D*_x_ = 1.281 Mg m^−^^3^
Orthorhombic, *P*2_1_2_1_2_1_	Mo *K*α radiation, λ = 0.71073 Å
Hall symbol: P 2ac 2ab	Cell parameters from 13225 reflections
*a* = 8.1643 (4) Å	θ = 3.0–27.4°
*b* = 9.9979 (4) Å	µ = 0.09 mm^−^^1^
*c* = 23.6503 (9) Å	*T* = 296 K
*V* = 1930.48 (14) Å^3^	Chunk, colorless
*Z* = 4	0.49 × 0.38 × 0.28 mm

### Data collection

**Table:**

Rigaku R-AXIS RAPID/ZJUG diffractometer	2183 independent reflections
Radiation source: rolling anode	1760 reflections with *I* > 2σ(*I*)
graphite	*R*_int_ = 0.030
Detector resolution: 10.00 pixels mm^-1^	θ_max_ = 26.0°, θ_min_ = 3.0°
ω scans	*h* = −10→10
Absorption correction: multi-scan (*ABSCOR*; Higashi, 1995)	*k* = −11→12
*T*_min_ = 0.956, *T*_max_ = 0.974	*l* = −29→29
16756 measured reflections	

### Refinement

**Table:**

Refinement on *F*^2^	Secondary atom site location: difference Fourier map
Least-squares matrix: full	Hydrogen site location: inferred from neighbouring sites
*R*[*F*^2^ > 2σ(*F*^2^)] = 0.034	H-atom parameters constrained
*wR*(*F*^2^) = 0.086	*w* = 1/[σ^2^(*F*_o_^2^) + (0.0289*P*)^2^ + 0.6417*P*] where *P* = (*F*_o_^2^ + 2*F*_c_^2^)/3
*S* = 1.00	(Δ/σ)_max_ < 0.001
2183 reflections	Δρ_max_ = 0.16 e Å^−^^3^
248 parameters	Δρ_min_ = −0.15 e Å^−^^3^
0 restraints	Extinction correction: *SHELXL97* (Sheldrick, 2008), Fc^*^=kFc[1+0.001xFc^2^λ^3^/sin(2θ)]^-1/4^
Primary atom site location: structure-invariant direct methods	Extinction coefficient: 0.0158 (10)

### Special details

**Table:**

Geometry. All e.s.d.'s (except the e.s.d. in the dihedral angle between two l.s. planes) are estimated using the full covariance matrix. The cell e.s.d.'s are taken into account individually in the estimation of e.s.d.'s in distances, angles and torsion angles; correlations between e.s.d.'s in cell parameters are only used when they are defined by crystal symmetry. An approximate (isotropic) treatment of cell e.s.d.'s is used for estimating e.s.d.'s involving l.s. planes.
Refinement. Refinement of *F*^2^ against ALL reflections. The weighted *R*-factor *wR* and goodness of fit *S* are based on *F*^2^, conventional *R*-factors *R* are based on *F*, with *F* set to zero for negative *F*^2^. The threshold expression of *F*^2^ > σ(*F*^2^) is used only for calculating *R*-factors(gt) *etc*. and is not relevant to the choice of reflections for refinement. *R*-factors based on *F*^2^ are statistically about twice as large as those based on *F*, and *R*- factors based on ALL data will be even larger.

### Fractional atomic coordinates and isotropic or equivalent isotropic displacement parameters (Å^2^)

**Table:**

	*x*	*y*	*z*	*U*_iso_*/*U*_eq_	
O3	0.5928 (2)	0.17729 (19)	0.38572 (8)	0.0590 (5)	
O1	0.3925 (2)	0.58802 (17)	0.41271 (7)	0.0552 (5)	
C9	0.3220 (3)	0.4541 (3)	0.32881 (10)	0.0448 (6)	
C10	0.3644 (3)	0.3262 (2)	0.36490 (10)	0.0452 (6)	
C4	0.4116 (3)	0.5769 (2)	0.35259 (9)	0.0467 (6)	
H4	0.5286	0.5683	0.3440	0.056*	
O4	0.8524 (3)	0.2138 (2)	0.35947 (10)	0.0769 (7)	
C17	0.7821 (3)	0.0043 (3)	0.40042 (10)	0.0481 (6)	
C6	0.2105 (4)	0.7223 (3)	0.30230 (11)	0.0516 (7)	
C8	0.1381 (3)	0.4869 (3)	0.33098 (11)	0.0529 (7)	
H8A	0.0763	0.4100	0.3179	0.063*	
H8B	0.1066	0.5044	0.3698	0.063*	
C5	0.3514 (4)	0.7072 (3)	0.32885 (11)	0.0514 (7)	
H5	0.4177	0.7821	0.3331	0.062*	
C18	0.6592 (4)	−0.0777 (3)	0.42095 (12)	0.0576 (7)	
H18	0.5513	−0.0480	0.4210	0.069*	
O2	0.2982 (3)	0.2494 (2)	0.46425 (8)	0.0710 (6)	
C7	0.0950 (4)	0.6076 (3)	0.29473 (12)	0.0607 (7)	
H7A	0.0952	0.5810	0.2553	0.073*	
H7B	−0.0149	0.6369	0.3041	0.073*	
C16	0.7495 (3)	0.1413 (3)	0.37958 (11)	0.0521 (7)	
C22	0.9427 (4)	−0.0412 (3)	0.40068 (11)	0.0565 (7)	
H22	1.0257	0.0137	0.3869	0.068*	
F1	0.8929 (3)	−0.36743 (17)	0.46149 (10)	0.1008 (7)	
C20	0.8560 (4)	−0.2447 (3)	0.44071 (14)	0.0665 (8)	
C2	0.5964 (4)	0.4062 (3)	0.42280 (13)	0.0663 (9)	
H2A	0.6725	0.4758	0.4117	0.080*	
H2B	0.6451	0.3541	0.4530	0.080*	
C13	0.1585 (4)	0.8535 (3)	0.27722 (11)	0.0645 (8)	
H13A	0.2345	0.9220	0.2882	0.097*	
H13B	0.0511	0.8762	0.2907	0.097*	
H13C	0.1564	0.8463	0.2367	0.097*	
C11	0.3138 (3)	0.3591 (3)	0.42485 (11)	0.0518 (7)	
C15	0.2899 (4)	0.1980 (3)	0.34125 (13)	0.0591 (7)	
H15A	0.2996	0.1277	0.3687	0.089*	
H15B	0.3469	0.1728	0.3074	0.089*	
H15C	0.1764	0.2126	0.3327	0.089*	
C14	0.3756 (4)	0.4322 (3)	0.26721 (11)	0.0636 (8)	
H14A	0.3711	0.5156	0.2472	0.095*	
H14B	0.3035	0.3690	0.2495	0.095*	
H14C	0.4855	0.3983	0.2665	0.095*	
C1	0.5533 (3)	0.3168 (3)	0.37240 (12)	0.0549 (7)	
H1	0.6103	0.3463	0.3381	0.066*	
C3	0.4327 (4)	0.4666 (3)	0.44166 (11)	0.0614 (8)	
H3	0.4312	0.4802	0.4827	0.074*	
C12	0.1571 (4)	0.3327 (3)	0.45174 (12)	0.0694 (8)	
H12A	0.1204	0.3945	0.4807	0.083*	
H12B	0.0706	0.2946	0.4287	0.083*	
C19	0.6964 (4)	−0.2039 (3)	0.44136 (14)	0.0680 (9)	
H19	0.6145	−0.2596	0.4552	0.082*	
C21	0.9811 (4)	−0.1672 (3)	0.42114 (13)	0.0651 (8)	
H21	1.0887	−0.1979	0.4215	0.078*	

### Atomic displacement parameters (Å^2^)

**Table:**

	*U*^11^	*U*^22^	*U*^33^	*U*^12^	*U*^13^	*U*^23^
O3	0.0414 (10)	0.0521 (10)	0.0836 (13)	−0.0025 (9)	0.0026 (10)	0.0180 (10)
O1	0.0717 (13)	0.0486 (9)	0.0453 (9)	−0.0096 (10)	−0.0136 (9)	0.0011 (8)
C9	0.0439 (15)	0.0497 (14)	0.0408 (12)	−0.0047 (12)	−0.0050 (11)	0.0007 (11)
C10	0.0418 (14)	0.0432 (13)	0.0505 (13)	−0.0070 (12)	−0.0056 (11)	0.0035 (11)
C4	0.0462 (15)	0.0498 (13)	0.0441 (12)	−0.0082 (13)	−0.0059 (11)	0.0058 (11)
O4	0.0477 (12)	0.0872 (16)	0.0958 (15)	−0.0058 (12)	0.0064 (11)	0.0384 (13)
C17	0.0456 (15)	0.0544 (15)	0.0443 (12)	−0.0032 (13)	−0.0004 (11)	−0.0004 (12)
C6	0.0571 (17)	0.0521 (16)	0.0457 (13)	0.0041 (14)	−0.0044 (13)	0.0033 (12)
C8	0.0462 (15)	0.0538 (15)	0.0587 (15)	−0.0060 (13)	−0.0093 (13)	−0.0004 (13)
C5	0.0588 (17)	0.0450 (14)	0.0505 (13)	−0.0086 (13)	−0.0078 (13)	0.0083 (11)
C18	0.0453 (16)	0.0545 (16)	0.0729 (17)	−0.0071 (14)	−0.0045 (14)	0.0025 (14)
O2	0.0814 (15)	0.0665 (12)	0.0651 (12)	−0.0101 (12)	0.0039 (11)	0.0243 (11)
C7	0.0522 (17)	0.0653 (17)	0.0647 (16)	0.0015 (15)	−0.0151 (14)	0.0030 (14)
C16	0.0431 (15)	0.0613 (16)	0.0518 (14)	−0.0041 (13)	−0.0025 (12)	0.0086 (13)
C22	0.0476 (16)	0.0649 (18)	0.0568 (15)	−0.0010 (14)	0.0076 (13)	0.0007 (14)
F1	0.0951 (15)	0.0492 (9)	0.1582 (19)	0.0028 (11)	−0.0263 (15)	0.0116 (11)
C20	0.068 (2)	0.0427 (14)	0.089 (2)	0.0002 (15)	−0.0128 (17)	−0.0025 (15)
C2	0.066 (2)	0.0576 (16)	0.0751 (18)	−0.0158 (16)	−0.0304 (16)	0.0173 (15)
C13	0.075 (2)	0.0609 (17)	0.0571 (15)	0.0136 (17)	−0.0039 (15)	0.0045 (14)
C11	0.0595 (17)	0.0507 (15)	0.0453 (13)	−0.0089 (14)	−0.0027 (12)	0.0101 (12)
C15	0.0565 (17)	0.0485 (15)	0.0723 (18)	−0.0081 (14)	−0.0036 (15)	−0.0055 (14)
C14	0.0688 (19)	0.0741 (19)	0.0481 (14)	0.0076 (18)	−0.0015 (14)	−0.0008 (14)
C1	0.0460 (15)	0.0491 (14)	0.0697 (16)	−0.0067 (13)	−0.0063 (14)	0.0159 (14)
C3	0.085 (2)	0.0547 (15)	0.0449 (13)	−0.0137 (17)	−0.0199 (15)	0.0086 (12)
C12	0.072 (2)	0.073 (2)	0.0625 (17)	−0.0079 (19)	0.0111 (16)	0.0079 (16)
C19	0.062 (2)	0.0494 (16)	0.093 (2)	−0.0131 (15)	−0.0047 (17)	0.0045 (15)
C21	0.0585 (18)	0.0628 (18)	0.0739 (18)	0.0097 (16)	0.0012 (15)	−0.0068 (16)

### Geometric parameters (Å, °)

**Table:**

O3—C16	1.337 (3)	C7—H7A	0.9700
O3—C1	1.466 (3)	C7—H7B	0.9700
O1—C3	1.432 (3)	C22—C21	1.386 (4)
O1—C4	1.435 (3)	C22—H22	0.9300
C9—C14	1.537 (4)	F1—C20	1.356 (3)
C9—C4	1.536 (3)	C20—C21	1.364 (4)
C9—C8	1.537 (4)	C20—C19	1.365 (5)
C9—C10	1.576 (3)	C2—C1	1.531 (4)
C10—C11	1.513 (4)	C2—C3	1.533 (5)
C10—C15	1.525 (3)	C2—H2A	0.9700
C10—C1	1.555 (4)	C2—H2B	0.9700
C4—C5	1.501 (3)	C13—H13A	0.9600
C4—H4	0.9800	C13—H13B	0.9600
O4—C16	1.207 (3)	C13—H13C	0.9600
C17—C18	1.384 (4)	C11—C12	1.452 (4)
C17—C22	1.388 (4)	C11—C3	1.502 (4)
C17—C16	1.480 (4)	C15—H15A	0.9600
C6—C5	1.319 (4)	C15—H15B	0.9600
C6—C7	1.496 (4)	C15—H15C	0.9600
C6—C13	1.501 (4)	C14—H14A	0.9600
C8—C7	1.522 (4)	C14—H14B	0.9600
C8—H8A	0.9700	C14—H14C	0.9600
C8—H8B	0.9700	C1—H1	0.9800
C5—H5	0.9300	C3—H3	0.9800
C18—C19	1.384 (4)	C12—H12A	0.9700
C18—H18	0.9300	C12—H12B	0.9700
O2—C11	1.445 (3)	C19—H19	0.9300
O2—C12	1.452 (4)	C21—H21	0.9300
			
C16—O3—C1	116.3 (2)	C1—C2—C3	104.8 (2)
C3—O1—C4	112.6 (2)	C1—C2—H2A	110.8
C14—C9—C4	109.0 (2)	C3—C2—H2A	110.8
C14—C9—C8	109.9 (2)	C1—C2—H2B	110.8
C4—C9—C8	106.4 (2)	C3—C2—H2B	110.8
C14—C9—C10	109.6 (2)	H2A—C2—H2B	108.9
C4—C9—C10	110.21 (18)	C6—C13—H13A	109.5
C8—C9—C10	111.7 (2)	C6—C13—H13B	109.5
C11—C10—C15	114.7 (2)	H13A—C13—H13B	109.5
C11—C10—C1	100.2 (2)	C6—C13—H13C	109.5
C15—C10—C1	112.7 (2)	H13A—C13—H13C	109.5
C11—C10—C9	105.7 (2)	H13B—C13—H13C	109.5
C15—C10—C9	113.3 (2)	O2—C11—C12	60.15 (17)
C1—C10—C9	109.2 (2)	O2—C11—C3	115.4 (2)
O1—C4—C5	105.6 (2)	C12—C11—C3	125.7 (3)
O1—C4—C9	111.87 (19)	O2—C11—C10	117.6 (2)
C5—C4—C9	113.6 (2)	C12—C11—C10	127.7 (2)
O1—C4—H4	108.6	C3—C11—C10	103.1 (2)
C5—C4—H4	108.6	C10—C15—H15A	109.5
C9—C4—H4	108.6	C10—C15—H15B	109.5
C18—C17—C22	119.3 (3)	H15A—C15—H15B	109.5
C18—C17—C16	122.4 (3)	C10—C15—H15C	109.5
C22—C17—C16	118.3 (2)	H15A—C15—H15C	109.5
C5—C6—C7	121.2 (2)	H15B—C15—H15C	109.5
C5—C6—C13	122.4 (3)	C9—C14—H14A	109.5
C7—C6—C13	116.4 (2)	C9—C14—H14B	109.5
C7—C8—C9	112.1 (2)	H14A—C14—H14B	109.5
C7—C8—H8A	109.2	C9—C14—H14C	109.5
C9—C8—H8A	109.2	H14A—C14—H14C	109.5
C7—C8—H8B	109.2	H14B—C14—H14C	109.5
C9—C8—H8B	109.2	O3—C1—C2	109.7 (2)
H8A—C8—H8B	107.9	O3—C1—C10	107.5 (2)
C6—C5—C4	124.3 (2)	C2—C1—C10	106.4 (2)
C6—C5—H5	117.9	O3—C1—H1	111.0
C4—C5—H5	117.9	C2—C1—H1	111.0
C19—C18—C17	120.2 (3)	C10—C1—H1	111.0
C19—C18—H18	119.9	O1—C3—C11	109.4 (2)
C17—C18—H18	119.9	O1—C3—C2	113.3 (2)
C11—O2—C12	60.18 (17)	C11—C3—C2	101.8 (2)
C6—C7—C8	113.3 (2)	O1—C3—H3	110.7
C6—C7—H7A	108.9	C11—C3—H3	110.7
C8—C7—H7A	108.9	C2—C3—H3	110.7
C6—C7—H7B	108.9	O2—C12—C11	59.67 (18)
C8—C7—H7B	108.9	O2—C12—H12A	117.8
H7A—C7—H7B	107.7	C11—C12—H12A	117.8
O4—C16—O3	123.2 (3)	O2—C12—H12B	117.8
O4—C16—C17	124.2 (3)	C11—C12—H12B	117.8
O3—C16—C17	112.6 (2)	H12A—C12—H12B	114.9
C21—C22—C17	120.9 (3)	C20—C19—C18	118.5 (3)
C21—C22—H22	119.6	C20—C19—H19	120.7
C17—C22—H22	119.6	C18—C19—H19	120.7
F1—C20—C21	118.1 (3)	C20—C21—C22	117.8 (3)
F1—C20—C19	118.6 (3)	C20—C21—H21	121.1
C21—C20—C19	123.3 (3)	C22—C21—H21	121.1
			
C14—C9—C10—C11	177.8 (2)	C12—O2—C11—C10	−119.7 (3)
C4—C9—C10—C11	57.9 (3)	C15—C10—C11—O2	38.5 (3)
C8—C9—C10—C11	−60.2 (3)	C1—C10—C11—O2	−82.5 (3)
C14—C9—C10—C15	−55.8 (3)	C9—C10—C11—O2	164.1 (2)
C4—C9—C10—C15	−175.7 (2)	C15—C10—C11—C12	−33.7 (4)
C8—C9—C10—C15	66.2 (3)	C1—C10—C11—C12	−154.7 (3)
C14—C9—C10—C1	70.8 (3)	C9—C10—C11—C12	91.9 (3)
C4—C9—C10—C1	−49.2 (3)	C15—C10—C11—C3	166.8 (2)
C8—C9—C10—C1	−167.2 (2)	C1—C10—C11—C3	45.8 (2)
C3—O1—C4—C5	175.3 (2)	C9—C10—C11—C3	−67.6 (2)
C3—O1—C4—C9	51.3 (3)	C16—O3—C1—C2	−81.3 (3)
C14—C9—C4—O1	−168.6 (2)	C16—O3—C1—C10	163.5 (2)
C8—C9—C4—O1	72.9 (3)	C3—C2—C1—O3	−117.3 (2)
C10—C9—C4—O1	−48.3 (3)	C3—C2—C1—C10	−1.4 (3)
C14—C9—C4—C5	72.0 (3)	C11—C10—C1—O3	90.8 (2)
C8—C9—C4—C5	−46.5 (3)	C15—C10—C1—O3	−31.6 (3)
C10—C9—C4—C5	−167.7 (2)	C9—C10—C1—O3	−158.49 (19)
C14—C9—C8—C7	−56.3 (3)	C11—C10—C1—C2	−26.7 (3)
C4—C9—C8—C7	61.5 (3)	C15—C10—C1—C2	−149.1 (2)
C10—C9—C8—C7	−178.2 (2)	C9—C10—C1—C2	84.0 (2)
C7—C6—C5—C4	1.4 (4)	C4—O1—C3—C11	−64.5 (3)
C13—C6—C5—C4	−177.5 (3)	C4—O1—C3—C2	48.2 (3)
O1—C4—C5—C6	−105.8 (3)	O2—C11—C3—O1	−157.9 (2)
C9—C4—C5—C6	17.1 (4)	C12—C11—C3—O1	−87.6 (3)
C22—C17—C18—C19	−0.2 (4)	C10—C11—C3—O1	72.5 (3)
C16—C17—C18—C19	−178.3 (3)	O2—C11—C3—C2	82.0 (3)
C5—C6—C7—C8	12.8 (4)	C12—C11—C3—C2	152.3 (3)
C13—C6—C7—C8	−168.2 (2)	C10—C11—C3—C2	−47.6 (2)
C9—C8—C7—C6	−45.5 (3)	C1—C2—C3—O1	−88.0 (2)
C1—O3—C16—O4	−6.6 (4)	C1—C2—C3—C11	29.3 (3)
C1—O3—C16—C17	173.1 (2)	C3—C11—C12—O2	−101.4 (3)
C18—C17—C16—O4	−177.1 (3)	C10—C11—C12—O2	103.4 (3)
C22—C17—C16—O4	4.7 (4)	F1—C20—C19—C18	179.0 (3)
C18—C17—C16—O3	3.3 (4)	C21—C20—C19—C18	0.3 (5)
C22—C17—C16—O3	−174.9 (2)	C17—C18—C19—C20	0.0 (5)
C18—C17—C22—C21	0.0 (4)	F1—C20—C21—C22	−179.1 (3)
C16—C17—C22—C21	178.2 (2)	C19—C20—C21—C22	−0.4 (5)
C12—O2—C11—C3	118.1 (3)	C17—C22—C21—C20	0.3 (4)
